# Enteric Virus Diversity Examined by Molecular Methods in Brazilian Poultry Flocks

**DOI:** 10.3390/vetsci5020038

**Published:** 2018-03-29

**Authors:** David I. De la Torre, Luis F. Nuñez, Claudete S. Astolfi-Ferreira, Antonio J. Piantino Ferreira

**Affiliations:** 1 Department of Pathology, School of Veterinary Medicine, University of São Paulo, São Paulo 05508-270, Brazil; daviddelatorreduque@gmail.com (D.I.D.l.T.); fabiann7@yahoo.es (L.F.N.); csastolfi@gmail.com (C.S.A.-F.); 2 School of Veterinary Medicine and Animal Science, Central University of Ecuador, Quito EC170521, Ecuador

**Keywords:** chicken, enteric virus, fowl adenovirus, astrovirus, avian nephritis virus, infectious bronchitis virus, reovirus, rotavirus

## Abstract

Enteric viruses play an important role in the Brazilian poultry industry due to the economic impact of resulting low yields of broilers, layers, and breeders. The most common enteric viruses affecting commercial flocks in Brazil include Fowl Adenovirus of group I (FAdV-I), Chicken Parvovirus (ChPV), Chicken Astrovirus (CAstV), Avian Nephritis Virus (ANV), Infectious Bronchitis Virus (IBV), Avian Reovirus (AReo), and Avian Rotavirus (ARtV). The aim of this study was to identify single and multiple infections using data obtained from 270 samples from eleven Brazilian states, corresponding to the period between 2010 and 2017. This was accompanied by an analysis of the relationship between the age of birds, clinical signs, and geographical distribution, using Polymerase Chain Reaction (PCR) and Reverse Transcription-PCR (RT-PCR) techniques. Twenty-five profiles of virus combinations were detected. Single infections were encountered in 86.3% of samples, and multiple infections were present in the remaining 13.7%. Both single and multiple infections affected all kinds of commercial chickens with digestive problems, stunting syndrome, decreases in egg and meat production, increased mortality, and respiratory signs. FAdV-I, ChPV, CAstV, ANV, and ARtV were mostly detected in young broilers, in contrast with IBV, which was detected in hens from one to greater than 51 weeks of age. These results exhibit the complexity of enteric diseases and the still poorly understood role of each pathogen as a unique etiological agent.

## 1. Introduction

Enteric viruses are the etiological agents for a series of health disturbances for commercial chickens around the world. They cause severe economic losses for the poultry industry because they negatively affect productive parameters, causing growth retardation, low feed consumption, high mortality, poor egg and meat production, and Runting-Stunting Syndrome (RSS) [1,2,3,4]. These kinds of infections affect mostly young birds, but it is common to find viral infections in birds of all ages, including broilers, layers, and breeders [5]. The main enteric viruses reported to cause enteric diseases are found in single and multiple infections and include the Fowl Adenovirus of group I (FAdV-I); Chicken Parvovirus (ChPV); two viruses from the *Astroviridae* family: Chicken Astrovirus (CAstV) and Avian Nephritis Virus (ANV); two viruses from the *Reoviridae* family: Avian Reovirus (AReo) and Avian Rotavirus (ARtV); and a member of the *Coronaviridae* family, Infectious Bronchitis Virus (IBV) [4,6,7,8,9]. Several laboratory analytical methods have been used to detect enteric viruses in organic tissues from sick and healthy birds. Conventional polymerase chain reaction (PCR) and reverse transcription-polymerase chain reaction (RT-PCR) are two of the most commonly used methods for diagnosis and characterization of viruses in the poultry industry [10,11,12,13,14]. The objective of this study was to determine the prevalence of enteric viruses affecting commercial chicken flocks in Brazil, encompassing an analysis of the relationships between single and multiple infections, the age of birds, clinical signs, and geographical distribution in the Brazilian states of Mato Grosso, Goias, Piaui, Ceara, Paraiba, Pernambuco, Bahia, Minas Gerais, Espirito Santo, São Paulo, and Santa Catarina. 

## 2. Materials and Methods

### 2.1. Sample Collection

For this study, we used 270 positive samples of single and multiple enteric viral infections from broilers, layers and breeders of different ages, collected from eleven Brazilian states: Mato Grosso, Goias, Piaui, Ceara, Paraiba, Pernambuco, Bahía, Minas Gerais, Espirito Santo, São Paulo, and Santa Catarina. The samples were collected between 2010 and 2017. Each sample was composed of a pool of maximum five organs of the same type, and each sample came from different farms across the mentioned states. The main reported symptoms of the birds were enteritis, diarrhea, decreased feed absorption, a decrease in production, mortality, signs of respiratory disease, and stunting syndrome. Samples were processed and analyzed by conventional PCR and RT-PCR for detection of seven main enteric viruses reported in Brazil: FAdV-I, ChPV, CAstV, ANV, IBV, AReo, and ARtV. The organs used for viral detection were the intestines, liver, pancreas, and caecal tonsils, and also cloacal swabs were used for fecal samples. The results were categorized according to the viral infection in each sample, the type of birds (broilers, layers, or breeders), the age of birds (days for broilers and weeks for layers and breeders), clinical signs, and origin of the samples based on their geographical distribution in Brazil. Known positive samples for FAdV-1, ChPV, CAstV, ANV, and ARtV, validated through Sanger sequencing, and commercial vaccines for IBV and AReo were used as positive controls.

### 2.2. DNA and RNA Extraction

The tissues were mechanically macerated and suspended in phosphate buffered saline (PBS), 0.1 M, pH 7.4, in a 1:1 proportion in 1500 µL microtubes. The mixture was vortexed, subjected to three freeze-thaw cycles, and subsequently clarified at 12,000× *g* for 20 min at 4 °C. An aliquot of each supernatant was used for DNA and RNA extraction, using the organic phenol-chloroform method, according to procedures described in [15,16], respectively. The concentration and quality of nucleic acids were evaluated in a NanoDrop 2000 (Thermo Fisher Scientific, Wilmington, DE, USA). Final DNA and RNA preparations were stored at −20 °C.

### 2.3. Reverse-Transcription Reaction

The samples screened for viruses with RNA genomes (CAstV, ANV, IBV, AReo, and ARtV), were submitted to a reverse transcriptase (RT) reaction to produce complementary DNA (cDNA), which was then used in the corresponding PCR. RT was conducted with the extracted RNA at a concentration of 3–5 µg/µL, which was denatured at 95 °C for 5 min and added to a mix containing 250 ng of Random primers, 500 µg/mL of Oligo (dT)_12_ primer, 10 mM of each deoxynucleotide triphosphates (dNTP), 4 µL of 5X First-Strand Buffer (250 mM Tris-HCl pH 8.3, 375 mM KCl, 15 mM Magnesium Chloride) (Invitrogen, Carlsbad, CA, USA), 2 µL of 100 mM Dithiothreitol (DTT) (Invitrogen), 200 units of Moloney Murine Leukemia Virus Reverse Transcriptase (M-MLV RT) (Invitrogen), and sterile distilled water, to reach 20 µL in final volume. The reaction was performed with the following temperature conditions: an initial incubation at 25 °C for 10 min, a second incubation at 37 °C for 50 min, and inactivation of the reaction at 70 °C for 15 min. RT products were stored at –20 °C. 

### 2.4. PCR for Fowl Adenovirus

The primers used for detection of FAdV-I were those described in [10], as shown in Table 1, and amplify an 897 bp segment corresponding to the variable region Loop 1 of the gene that encodes the structural protein, Hexon. Through analysis of the nucleotide and amino acid sequences, it is possible to characterize the 12 different serotypes of FAdV-I (FAdV-1–FadV-8a, FAdV-8b–FAdV-11) [10]. DNA amplification was carried out in 200 µL microtubes by adding 2.5 µL of extracted DNA with a concentration of 1 µg/µL to a mix containing 1X PCR magnesium free (−Mg) Buffer (Invitrogen), 1.25 mM of each deoxynucleotide triphosphate, 0.5 µM of each primer, (Hexon A and Hexon B), 1 U of Platinum^TM^ Taq DNA Polymerase, 1.5 mM of MgCl_2_ (Invitrogen), and sterile distilled water, to reach 22.5 µL. The following temperature conditions were used for the PCR reactions: a cycle of 94 °C for 5 min, 35 cycles at 94 °C for 60 s, 52 °C for 45 s, and 72 °C for 1 min, followed by a final extension at 72 °C for 10 min.

### 2.5. PCR for Chicken Parvovirus

The primers used for detection of ChPV were those described in [17] (Table 1) and amplify a 561 bp segment corresponding to a well-conserved region of the nonstructural gene (NS) usually used for detection of chicken and turkey parvovirus [17]. DNA amplification was carried out in 200 µL microtubes by adding 2 µL of extracted DNA with a concentration of 1 µg/µL into a mix containing 1X PCR magnesium free (−Mg) Buffer, 1.25 mM of each deoxynucleotide triphosphate, 0.5 µM of each primer (PVF1 and PVR1), 1.25 U of Platinum^TM^ Taq DNA Polymerase, 2 mM of MgCl_2_, and enough sterile distilled water to reach 23 µL. The following temperature conditions were used for the PCR reaction: a cycle of 94 °C for 3 min, 35 cycles at 94 °C for 30 s, 55 °C for 30 s, 72 °C for 1 min, followed by a final extension at 72 °C for 10 min.

### 2.6. RT-PCR for Chicken Astrovirus and Avian Nephritis Virus

PCR was conducted with the cDNA produced by the RT reaction, using the primers described in [12] (Table 1). This was used for the diagnosis of CAstV and ANV, which results in the amplification of 362 bp and 473 bp segments, respectively, both of which are located in the RNA polymerase gene (ORF 1B) [12]. DNA amplification was done in 200 µL microtubes by adding 2.5 µL of cDNA to a mix containing 1X PCR magnesium free (−Mg) Buffer, 1.25 mM of each deoxynucleotide triphosphate, 0.5 µM of each primer (Cas pol 1F and Cas pol 1R for CAstV; ANV pol 1F and ANV pol 1R for ANV), 1 U of Platinum^TM^ Taq DNA Polymerase, 1.5 mM of MgCl_2_, and enough sterile distilled water to reach 22.5 µL. The following temperature conditions were used for the PCR reaction: a cycle at 95 °C for 5 min, 35 cycles at 94 °C for 30 s, 50 °C for 30 s, 72 °C for 1 min, and a final extension at 72 °C for 10 min. 

### 2.7. RT-PCR for Coronavirus

PCR was conducted with the cDNA produced by the RT reaction, using the primers for detection of all known coronaviruses, described in [11] (Table 1), which amplifies a 266 bp segment located in a highly conserved region of the 3′-untranslated region (3′-UTR) in the first PCR reaction (UTR-11 and UTR-41) and a 179 bp segment in a second, hemi-nested reaction (UTR-31 and UTR-41) [11]. Amplification of the first and second PCR reactions was performed in 200 µL microtubes by adding 2.5 µL of cDNA in the first PCR reaction and 1 µL of the PCR product for the second reaction. The mix for the two PCR reactions contained 1X PCR magnesium free (−Mg) Buffer, 1.25 mM of each deoxynucleotide triphosphate, 0.5 µM of each primer (UTR-11, UTR-31 and UTR-41), 1.25 U of Platinum^TM^ Taq DNA Polymerase, 2 mM of MgCl_2_, and enough sterile distilled water to reach 22.5 µL for the first PCR reaction and 24 µL for the second PCR hemi-nested reaction. The following temperature conditions were used for both PCR reactions: a cycle at 94 °C for 3 min, 35 cycles at 94 °C for 1 min, 48 °C for 90 s, 72 °C for 90 s, and a final extension at 72 °C for 10 min. 

### 2.8. RT-PCR for Avian Rotavirus

PCR was conducted with the cDNA produced by the RT reaction, using the primers described in [12] (Table 1), which amplify a 630 bp segment located in Segment 10 (S10) of the viral genome, encoding the non-structural protein, NSP4 [12]. DNA amplifications were conducted in 200 µL microtubes by adding 2.5 µL of cDNA to a mix containing 1X PCR magnesium free (−Mg) Buffer, 1.25 mM of each deoxynucleotide triphosphate, 0.5 µM of each primer (NSP4-F30 and NSP4-R660), 1 U of Platinum^TM^ Taq DNA Polymerase, 1.5 mM of MgCl_2_, and enough sterile distilled water to reach 22.5 µL. The following temperature conditions were used for the PCR reaction: a cycle at 95 °C for 5 min, 35 cycles at 94 °C for 30 s, 50 °C for 30 s, 72 °C for 1 min, and a final extension at 72 °C for 10 min.

### 2.9. RT-PCR for Avian Reovirus

PCR was conducted with the cDNA produced by the RT reaction, using the primers described in [13] (Table 1), which amplify a 1120 bp fragment located in Segment 4 (S4) of the viral genome, encoding the structural protein 4 (VP4) [13]. DNA amplifications were conducted in 200 µL microtubes by adding 5 µL of cDNA to a mix containing 1X PCR magnesium free (−Mg) Buffer, 1.25 mM of each deoxynucleotide triphosphate, 0.5 µM of each primer (S4-F13 and S4-R1133), 1.25 U of Platinum^TM^ Taq DNA Polymerase, 2 mM de MgCl_2_, and enough sterile distilled water to reach 20 µL. The following temperature conditions were used for the PCR reaction: a cycle at 94 °C for 5 min, 35 cycles of 94 °C for 30 s, 50 °C for 1 min, 72 °C for 1 min, and a final extension at 72 °C for 10 min.

### 2.10. Evaluation of RT-PCR and PCR Products

RT-PCR and PCR products were analyzed by electrophoretic diffusion in 1.5% Ultrapure^TM^ Agarose (Invitrogen) gels submerged in 0.5X Tris-borate-Ethylenediamine Tetraacetic acid (EDTA). The size of DNA fragments was estimated by comparison with the 100 bp DNA Ladder (Invitrogen). A UV trans-illumination camera was used to visualize the DNA bands.

### 2.11. Sequencing and Phylogenetic Analysis

Some samples were chosen randomly for DNA sequencing to validate the RT-PCR- and PCR-specific reactions: FAdV-I (*n* = 3), ChPV (*n* = 3), CAstV (*n* = 3), ANV (*n* = 4), AReo (*n* = 1), and ARtV (*n* = 3). The nucleotide sequences for each sample were submitted to GenBank with the exception of the FAdV-I and ChPV sequences, which had already been submitted in previous studies. IBV sequences were not analyzed phylogenetically due to the high diversity of coronaviruses in different animal species. PCR products from each sample were purified using the GPX™ PCR DNA and Gel Band Purification kit (GE Healthcare, Piscataway, NJ, USA), following the manufacturer’s instructions. Purified products were sequenced in the forward and reverse directions using the BigDye^®^ Terminator Cycle Sequencing Kit v3.1 (Invitrogen) based on the manufacturer’s instructions. Sequenced products were analyzed in an ABI 3730 DNA Analyzer (Applied Biosystems by Life Technologies, Carlsbad, CA, USA). Electropherograms obtained from the sequencing process were edited in the CLC Main WorkBench 7.7.3 (CLC Bio-Qiagen, Aarhus, Denmark) software and aligned with the Clustal W method available in the ClustalX 2.1 software (Des Higgins Conway Institute, UCD, Dublin, Ireland), using reference sequences from the NCBI GenBank for each enteric virus with the following access codes: FAdV-I: FAdV-1 (NC_001720), FAdV-2 (AF339915), FAdV-3 (AF508949), FAdV-4 (NC_015323), FAdV-5 (NC_021221), FAdV-6 (AF508954), FAdV-7 (AF508955), FAdV-8a (KT862810), FAdV-8b (JN112373, KT862811, KX258422, KU981150, and KU981150), FAdV-9 (NC_000899), FAdV-10 (KT717889), and FAdV-11 (AF339925); ChPV: (KU569462, GQ281296, KJ486491, JQ178304, KT347548, KX133426, HQ680340, NC_024452, JF267323, KM598417, and KY649278); CAstV: (KC633180, JX945871, KT386328, KX397575, JF414802, and DQ324839); ANV: (DQ324833, KM254166, KM985702, HM029238, and NC_003790); ARtV: (KX185128, JX474761, GQ353331, KT347547, EU400310, JQ085408, LC088136, FJ169862, and LK932176); AReo: (KX398291, KC865795, JQ954693, EU400285, KP173692, JN641885, JN641884, KF741745, and DQ198858). Phylogenetic trees were inferred using the neighbor-joining statistical method, with 1000 bootstraps replications, integrated in the MEGA 7 software (Pennsylvania State University, PA, USA).

### 2.12. Statistical Analysis

Descriptive statistics were used to represent the variability of the positive samples, using the single and multiple viral infections, type of birds (broilers, layers and breeders), age of birds (days for broilers and weeks for layers and breeders), clinical signs (respiratory signs, digestive signs, stunting-mortality-drop production, and no clinical signs), and geographic distribution of the samples. Minitab 18 (Minitab^®^ Statistical Software, v18.1, Minitab Inc., State College, PA, USA) was used to develop the variance analysis of frequencies of single and multiple infections for each virus, and clinical signs pertaining to each type of bird. The alfa value for significance level was α = 0.05.

## 3. Results

### 3.1. Single and Multiple Viral Infections

A total of 333 viruses were found in 270 samples, and the most common virus detected corresponded to IBV, showing a 58.9% (196/333) occurrence, followed by ANV with 12.6% (42/333), FAdV-I with 8.4% (28/333), CAstV with 8.1% (24/333), ChPV with 6.6% (22/333), ARtV with 5.1% (17/333), and AReo with 0.3% (1/333). Single infections were found in 86.3% (233/270) of the samples, with IBV being the most predominant virus diagnosed as a unique agent. ANV, CAstV, IBV, ChPV, and ARtV were found in simple and combined (2 to 5 viruses) infections. FAdV-I was found in unique and combined (2 to 3 viruses) infections. Multiple infection analysis showed that 2 viral infections were found in 6.3% (17/270) of samples, followed by 3 viral infections in 5.6% (15/270), 4 viral infections in 1.5% (4/270), and 5 viral infections in 0.4% (1/270). Single and multiple viral infections are described in Table 2. Multiple infections occurred with 25 profile combinations of viruses (Table 3). ANV, CAstV, IBV, ChPV, and ARtV were present in combination with each other, resulting in between two and five virus combinations. FAdV-I was present in combination with ChPV, ANV, and IBV. AReo showed a single infection. The variance analysis showed no significant difference between the frequencies of single and multiple viruses (*p* = 0.36).

### 3.2. Age and type of birds

The samples received for viral diagnostics were from broilers, layers, and breeders of all ages. According to the age of birds, the samples corresponding to layer and breeder hens were divided into 5 groups, with intervals of 10 weeks of age, from week 1 to week 50, and two more groups, one for birds older than 51 weeks, and a group for which the ages were not reported (N/R; Table 4). 

The samples corresponding to broilers were divided into 6 groups with intervals of 7 days of age, from day 1 to day 42, and two more groups, one for birds older than 43 days, and an N/R age group (Table 5). 

Positive samples from layer and breeder hens older than 51 weeks of age showed the highest frequency of viral infections: 22.4% for layers and 37.8% for breeders. The viral infection frequencies of the other layer groups were 18.4% for the 11–20-week group, 16.3% for the 1–10-week and 31–40-week groups, 4.1% for the 21–30-week group, and 22.4% for the N/R age group. In the case of breeders, the frequency of viral infections was 18.9% for the 1–10-week and 41–50-week groups, 13.5% for the 31–40-week group, and 10.8% for the N/R age group (Table 4). Broilers showed the highest frequency of viral infections, at 37.8% for the 36–42-day group. Viral infection rates in the other groups were 15.3% for the 8–14-day group, 14.7% for the 15–21-day group, 11.7% for the 43-day group, 7.4% for the 1–7-day group, 6.7% for the 22–28-day group, 4.9% for the 29–35-day group, and 6.7% for the N/R age group (Table 5). Single viral infections were found mostly in broilers, with 129/233 (55.4%), followed by 49/233 (21%) in layers and 34/233 (14.6%) in breeders. Multiple viral infections were found with more frequency in broilers, with 14/17 (82.4%) with two viruses, 15/15 (100%) with three viruses, and 4/4 (100%) with four viruses. The only sample with five viral infections also belonged to the broilers group. A total of 3/17 (17.6%) samples with multiple viral infections (3 viruses) were found in samples from breeders (Table 6). 

The organs corresponding to positive results for FAdV, ChPV, CAstV, and ARtV included the liver, intestines, and pancreas. AReo was present in one sample of intestines and IBV was present in all the five organs used for this study (Table 7).

FAdV-I was found in samples from breeders (12/28) and broilers (14/28). ChPV was found in samples from broilers (20/22). CAstV was found in samples from breeders (10/27) and broilers (17/27). ANV was found in samples from broilers (41/42). IBV was found in samples from layers (49/196), breeders (16/196), and broilers (116/196). AReo was found in one sample, but the origin was not reported. ARtV was found in samples from breeders (2/17) and broilers (15/17). All data describing the positive samples according to the age of layers, breeders, and broilers affected by the enteric viruses in this study are shown in Table 8. 

### 3.3. Clinical Signs

Positive samples were divided into five groups according to clinical signs and type of birds. Respiratory signs were reported in 39/270 samples, digestive signs in 9/270, stunting-mortality-decreased production signs in 69/270, no clinical signs in 5/270, and the remaining 148/270 samples did not include reports regarding clinical signs. The most common disease symptoms reported were stunting syndrome, mortality, and a decrease in egg and meat production, which occurred in 69/270 (25.6%) of the positive samples. Broilers showed the highest values of stunting-mortality-decrease in production, with 55/69 (79.7%), followed by breeders with 12/69 (17.4%) and layers with 2/69 (2.9%). An important factor to be considered is that respiratory pathogens causing respiratory problems were found in digestive organs, for example IBV, with 12/39 (30.8%) positive samples in broilers, 23/39 (59%) in layers, and 3/39 (7.7%) in breeders. Digestive diseases were not commonly reported as having unique clinical signs, and only 9 positive samples were obtained from broilers. All frequencies of clinical signs are described in Table 6. The variance analysis showed no significant difference between clinical signs for each type of bird (*p* = 0.45).

### 3.4. Geographic Origin

Samples originated from 12 Brazilian states from the north-east, east, and south-east regions. Most of the samples positive for enteric viruses were from to the state of São Paulo 82/270 (30.4%). IBV was the main virus found in 11 states, accounting for 133/333 of the total viruses detected, excluding the state of Paraiba. FAdV-I was detected in samples originating from Pernambuco, Santa Catarina, and São Paulo, with a total of 26/333 virus detected. ChPV and ANV were detected in samples from Bahía, Ceara, Minas Gerais, Paraiba, Pernambuco, and São Paulo, with a total of 15/333 and 37/333 detections, respectively. CAstV and ARtV were detected in the state of Minas Gerais with 20/333 and 15/333 virus detected, respectively, though ARtV was also present in 1 detection of Pernambuco. The origin of the remaining viruses detected, i.e., FAdV-I (2/333), ChPV (7/333), CAstV (7/333), ANV (5/333), IBV (63/333), AReo (1/333), and ARtV (2/333), were not reported. Data describing the geographical distribution of enteric viruses are shown in Table 9. 

### 3.5. Phylogenetic Analysis

**Figure 1:**
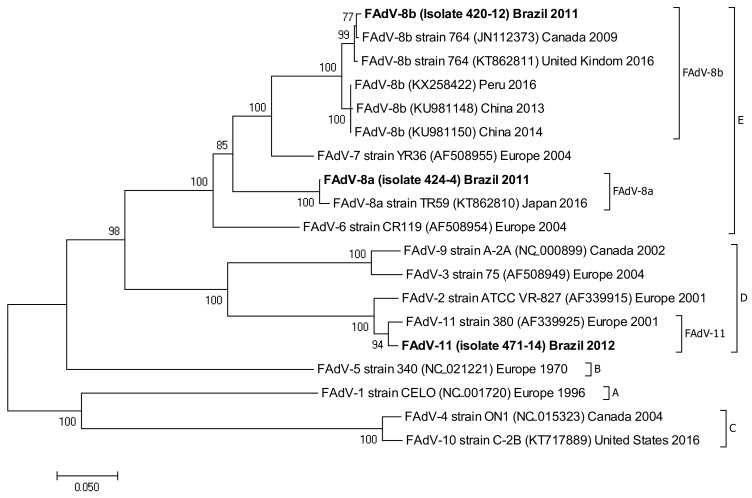
Phylogenetic tree for FAdV-I strains, was inferred with the neighbor-joining statistical method and based on the partial sequence of the Hexon gene. Numbers along the branches refer to bootstrap values for 1000 replicates. The five species of FAdV-I are grouped and identified with letters A–E.

The following accession numbers correspond to the sequences used in the phylogenetic analysis: FAdV-I: isolate 420-12 (KY229185), isolate 424-4 (KY229176), and isolate 471-14 (KY229184); ChPV: isolate 691-1 (MF784849), isolate 752-3 (MF784850), and isolate 786-1 (MF784851); ANV: isolate 475-5 (MF683400), isolate 752-3 (MF683401), isolate 656-3 (MF683402), and isolate 691-6 (MF683403); CAstV: isolate 541-2 (KR013275), isolate 541-12 (KR013255), and isolate 541-16 (KR013252); ARtV: isolate 480-1 (MF683404), isolate 480-7 (MF683405), and isolate 480-5 (MF683406); AReo: isolate 806-1 (MF693911). FAdV-I and CAstV sequences were already submitted to the GenBank database in previous studies [18]. Fowl adenovirus sequences were characterized according to their position in the phylogenetic tree (Figure 1). 

**Figure 2:**
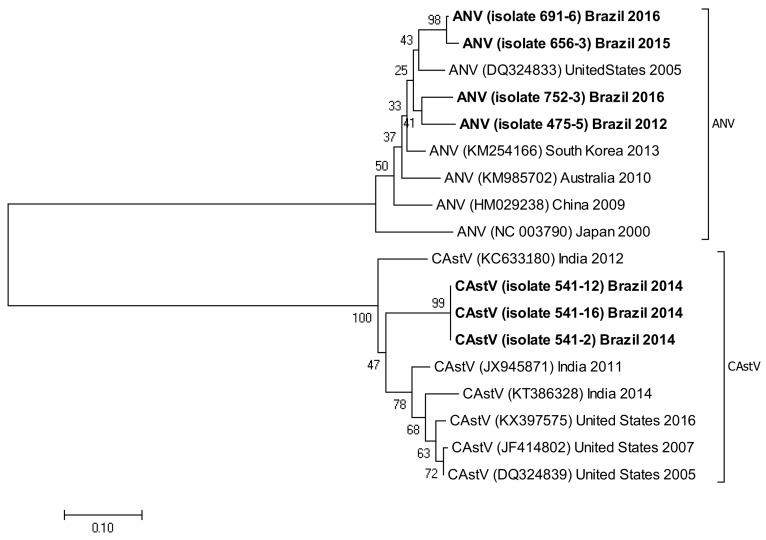
Phylogenetic tree for ANV and CAstV strains, was inferred with the neighbor-joining statistical method and based on the partial sequence of the ORF-1b gene in both of them. Numbers along the branches refer to bootstrap values for 1000 replicates.

Isolate 424-4 was clustered with the reference sequence of strain TR59 with a bootstrap value of 100 in the common ancestral line, suggesting that this isolate should be classified into the FAdV-8a serotype. Isolate 420-12 was clustered with reference sequences of strain 764, and some isolates originated from China and Peru, with a bootstrap value of 100 in the common ancestor of the 6 sequences, suggesting that this isolate should be classified as FAdV-8b serotype. Isolate 471-14 clustered with the reference sequence of strain ATCC, suggesting that this isolate should be classified as FAdV-11 serotype. Isolates 475-5, 656-3, 691-6, and 752-3, clustered with reference sequences of Avian Nephritis Virus from the United States, Korea, Australia, China, and Japan, and a second group of isolates (541-2, 541-12, and 541-16) were clustered with reference sequences of chicken astrovirus from India and the United States, showing high bootstrap values for each group of both types of astroviruses detected in this study (Figure 2). 

**Figure 3:**
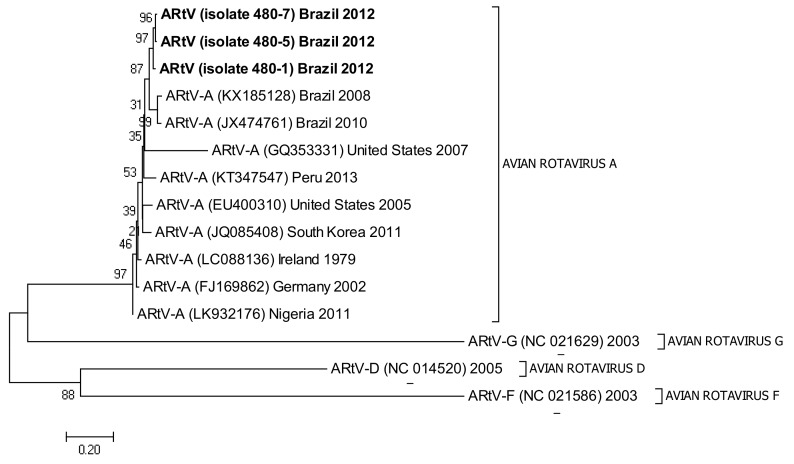
Phylogenetic tree for ARtV strains, was inferred with the neighbor-joining statistical method and based on the partial sequence of the NSP4 gene. Numbers along the branches refer to bootstrap values for 1000 replicates. Sequences of ARtV G, D, and F were used as out-group controls.

The three sequences that were positive for ARtV were analyzed with reference sequences from the United States, Peru, South Korea, Ireland, Germany, and Nigeria, all of them clustering with a high bootstrap value with the common ancestor. Reference sequences for groups D, F, and G rotavirus were used as external groups to show the differentiation of the positive samples from Avian Rotavirus A (Figure 3). 

**Figure 4:**
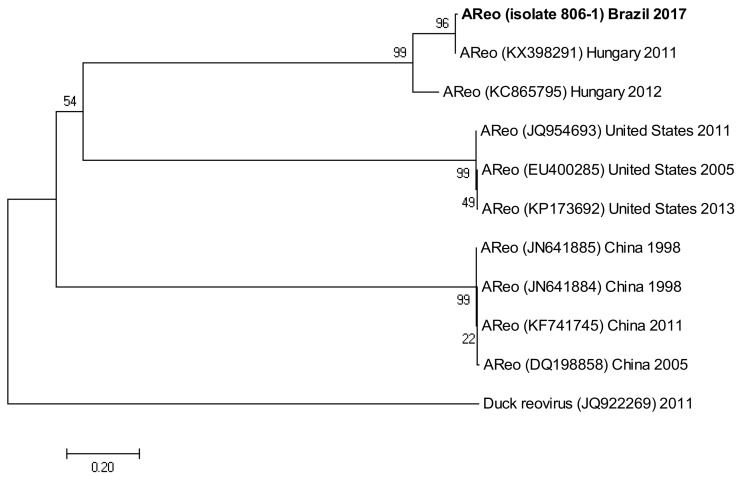
Phylogenetic tree for AReo strains, was inferred with the neighbor-joining statistical method and based on the partial sequence of segment S4. Numbers along the branches refer to bootstrap values for 1000 replicates. Sequence of Duck Reovirus was used as out-group control.

Isolate 806-1 clustered with reference sequences of AReo from Hungary. The branch lengths and low bootstrap values from the common ancestor for reference sequences from Hungary, the United States, and China, all show the magnitude of the genetic change among different isolates from these countries (Figure 4). 

**Figure 5:**
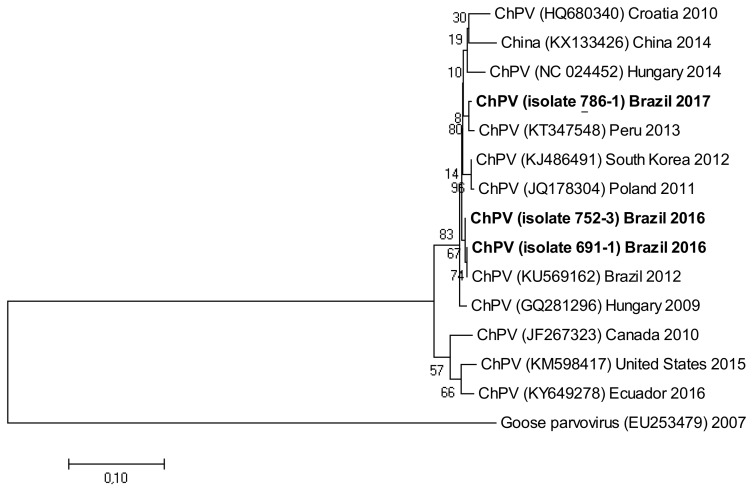
Phylogenetic tree for ChPV strains, was inferred with the neighbor-joining statistical method and based on the partial sequence of NS gene. Numbers along the branches refer to bootstrap values for 1000 replicates. Sequence of Goose parvovirus was used as out-group control.

The three isolates corresponding to ChPV were analyzed phylogenetically with strains from different countries from America, Europe, and Asia (Figure 5).

Isolate 786-1 clustered with an isolate of ChPV from Peru, whereas isolates 752-3 and 691-1 clustered in a different group, together with Brazilian isolates, but remained in the main group with all isolates of chicken parvovirus. Goose parvovirus was used in this tree as an external group. The phylogenetic trees were inferred using the neighbor-joining method [19]. The percentage of replicate trees in which the associated taxa clustered together in the bootstrap test (1000 replicates) are joined to the branches [20]. The tree was drawn to scale, with branch lengths in the same units as the evolutionary distances used to infer the phylogenetic tree. The evolutionary distances were computed using the Tamura-Nei method [21] and are in units of the number of base substitutions per site.

## 4. Discussion

Many etiological agents, such as bacteria, fungi, parasites, mycotoxins, and viruses, are related to enteric diseases. Enteric viruses have been considered the main causative etiological agents of RSS and acute enteric disturbances that negatively affect the yield of commercial chickens [1,2,4,22,23]. The most common viruses associated with the enteric diseases of chickens and turkeys include FAdV-I, ChPV, CAstV, ANV, IBV, AReo, and ARtV [7,9]. Clinical signs reported for each sample used in this study were related to digestive problems (diarrhea and feed intake), respiratory signs, stunting, high mortality, and a decrease in egg and meat production, which is consistent with results for clinical signs of enteric viruses obtained in other studies around the world [3,4,7,24,25,26,27]. Molecular techniques such as PCR and nucleotide sequencing were employed due to their wide use in the diagnosis of enteric viruses [9,22,28]. The association of enteric virus with the age of broilers, breeders, and layers (Table 4 and Table 5) showed that molecular diagnosis of these viruses can be performed at different stages of production, which can be useful in the control of vertical infections. For example, in our results, ChPV, ANV, and IBV were present in broilers in the first week of age (Table 8), constituting possible vertical transmission. A high number of enteric viruses were found in birds from all ages and in different stages of production, both in broilers and hens (Table 4 and Table 5). Enteric viral infections mostly affect young birds, probably due to their immature intestine epithelium, which is the first organ affected during a viral infection. This results in poor feed absorption with the consequent general symptoms [5]. FAdV-I was detected in young and old birds (Table 4 and Table 5), corroborating results obtained previously [29], where the virus was found in several samples from broilers, layers, and domestic chickens of all ages, even though the clinical signs were more severe in younger birds [30]. ChPV infections affect young birds in their first four weeks [23,27], which is supported by our findings since the virus was detected in 20 infected samples from broilers between 1 and 28 days old (Table 8). AReo was found in 15/333 detections, corresponding to broilers in the growth phase (Table 8), especially between 8 to 28 days old, showing that rotavirus infections mainly affect young birds, promoting RSS and enteric lesions [1]. Both astroviruses, CAstV and ANV, were found in samples from young broilers, despite having nine positive samples from breeders older than 51 weeks old (Table 8). Astrovirus infections usually affect young birds [24], although these viruses can be found in older birds in single and multiple infections with other enteric viruses [8]. IBV affects birds of virtually all ages [31] as demonstrated by our results, where samples of broilers, layers, and breeders from one week and greater than 51 weeks (Table 8) presented positive results for coronavirus in all digestive organs tested, despite the fact that some clinical signs reported for these samples were respiratory signs. Enteric viruses such as FAdV-I, ChPV, CAstV, ANV, IBV, and ARtV were present in the liver, intestines, and pancreas. Caecal tonsils and cloacal swabs were used exclusively for diagnosis of IBV (Table 7). Viral particles of FAdV-I were found in the liver, intestines, and pancreas. The organs where this virus can be detected are the liver, intestines, caecal tonsils, proventriculus, bursa of Fabricius, thymus, spleen, lungs and kidneys [32,33,34,35], especially in the first week of infection. Based on the pancreatic tissue samples that were positive for FAdV-I (5/28) (Table 7), we confirmed the importance of this organ for diagnosis of diseases caused by fowl adenovirus and even in cases of pancreatitis [36]. ChPV was detected in the liver, intestines, and pancreas, which are areas commonly used for ChPV detection due its pathogenicity as an enteric virus, considering the variety of organs where this virus can be found, including the brain, duodenal loop, and even on cloacal swabs [27,37,38]. Astroviruses, such as CAstV and ANV, are commonly detected in the digestive organs and fecal samples of sick and healthy birds [39,40,41], supporting our findings of astrovirus in the liver, intestines, and pancreas (Table 7). IBV can be detected in the intestinal content, trachea, lung, liver, bursa of Fabricius, pancreas, thymus, kidney, proventriculus, and spleen of affected birds [13,42], according to the dynamic distribution of the virus, resulting in a wide range of useful organs for molecular detection of this virus, which in our case helped to determine the presence of the pathogen in liver, intestine, pancreas, cloacal swabs, and caecal tonsils (Table 7). In our study, we found three different serotypes of FAdV-I, without ruling out the possibility of multiple FAdV infections in the same sample because multiple strains of different serotypes can affect to the same animal [29]. Multiple infections between FAdV-I and other enteric viruses were found in our study, especially with ChPV, ANV, and IBV, supporting previous results showing that FAdV-I is often present in co-infections with other enteric viruses including CAstV, AReo, and ARtV [7]. ChPV was present in co-infections with one to four viruses, including IBV, ANV, FAdV-I, and CAstV, which has also been reported in metagenomic [3] and epidemiological surveys [38] with broilers showing RSS. The most common virus found in this study belongs to the family *Coronaviridae*, which indicates the presence of Infectious Bronchitis Virus in the processed samples. A high percentage of positive samples (60.7%) (Table 3.) indicated the presence of single IBV infections, probably due to the absence of co-infections with other enteric viruses or because only IBV analysis was requested. IBV co-infections with multiple enteric viruses were demonstrated in 32 samples (Table 2), making it difficult to define the exact role of IBV in enteric diseases [43], especially in multiple viral infections. All of the enteric viruses detected during 2010 and 2017 are widely distributed around the world. FAdV-I, found in this study, is a common virus, widely distributed in Brazil and well-reported throughout the world [9,13,44,45,46,47,48], demonstrating its wide distribution among commercial birds. The phylogenetic tree showed that ChPV has a well-conserved genome in non-structural genic regions, as evidenced by the branch sizes in comparison with reference sequences from GenBank, further demonstrating the distribution of ChPV around the world [14,49,50]. Astroviruses affecting commercial flocks are also widely distributed worldwide [28,39,51]. Phylogenetic analysis of astroviruses clustered ANV and CAstV in two different groups, and each of them was sub-grouped with the nucleotide sequences of the RNA polymerase gene from isolates originating in North-America and Asia, providing a resource to determine the global distribution of these viruses. The ARtV analyzed in this study showed a close phylogenetic relationship with isolates from the United States, Peru, South Korea, Ireland, Germany, Nigeria, and Brazil, particularly in the length of the branches in the phylogenetic tree, which may confirm the complete distribution of the virus around the world [22,24,52,53,54]. Coronaviruses such as IBV are some of the most widely distributed enteric viruses in Brazil and have attracted special interest from the poultry industry due the economic losses caused by its impact on the health of birds [55,56,57]. The geographical distribution of the enteric viruses analyzed in this study was reported previously in some regions from the Brazilian territory. According to this study, FAdV-I was disseminated in the states of Paraiba, Pernambuco, São Paulo, and Santa Catarina, in addition to previously reported isolates of FAdV-I and ChPV from the state of Rio Grande do Sul [58]. CAstV was found in samples from Minas Gerais, and the other member of the family, *Astroviridae* (ANV), was found in samples from Ceara, Paraiba, Pernambuco, Bahia, Minas Gerais, and São Paulo, increasing the previously reported distribution of astroviruses in the states of Rio Grande do Sul, Parana, and Mato Grosso [59,60]. ChPV was distributed in the states of Ceara, Paraiba, Pernambuco, Bahia, Minas Gerais, and São Paulo, supporting the results of [38], who reported the presence of this virus in an outbreak in São Paulo, and expanding upon the report of [27], who worked with isolates of ChPV from poultry farms in the state of Rio Grande do Sul. The ARtV results suggest that this virus was disseminated in the states of Pernambuco, Minas Gerais, and São Paulo, consistent with other previous reports that describe the presence of ARtV in the states of Minas Gerais, Parana, Rio Grande do Sul, and Goias [61,62]. IBV is the most disseminated virus among Poultry flocks in Brazil [63]. This virus requires more specific analysis for the characterization of the different genotypes that affect commercial chickens in Brazilian states and other countries in South America, to determine the epidemiological dynamics and pathogenesis of the virus within the poultry industry. 

## 5. Conclusions

These results may contribute to understand the geographical distribution of enteric viruses within Brazilian territory and the worldwide epidemiology through the evolution pattern shown in the current phylogenetic analysis, providing useful data to be used in future studies. 

## Figures and Tables

**Table 1:** Enteric viruses screened in this study with the name of the primers used in the RT-PCR and PCR reactions. Primer sequences and amplicon sizes are expressed in base pairs (bp).

Virus	Gene Target *	Primer Name	Primer Sequence	bp	Reference
FAdV-I	Hexon	Hexon A	5’-CAARTTCAGRCAGACGGT-3’	897	[10]
		Hexon B	5’-TAGTGATGMCGSGACATCAT-3’		
ChPV	NS	PVF1	5’-TTCTAATAACGATATCACTCAAGTTTC-3’	561	[17]
		PVR1	5’-TTTGCGCTTGCGGTGAAGTCT GGCTCG-3’		
IBV	UTR	UTR 11	5’-GCTCTAACTCTATACTAGCCTA-3’	179	[11]
		UTR 31	5’-GGGCGTCCAAGTGCTGTACCC-3’		
		UTR 41	5’-ATGTCTATCGCCAGGGAAATGTC-3’		
CAstV	ORF 1b	CAS pol 1F	5’-GAYCARCGAATGCGRAGRTTG-3’	362	[12]
		CAS pol 1R	5’-TCAGTGGAAGTGGGKARTCTAC-3’		
ANV	ORF 1b	ANV pol 1F	5’-GYTGGGCGCYTCYTTTGAYACCRT-3’	473	[12]
		ANV pol 1R	5’-CRTTTGCCCKRTARTCTTTRTGAY-3’		
AReo	S4	S4-F13	5’-GTGCGTGTTGGAGTTTCCCG-3’	1,120	[13]
		S4-R1133	5’-TACGCCATCCTAGCTGGA-3’		
ARtV	NSP4	NSP4-F30	5’-GTGCGGAAAGATGGAGAAC-3’	630	[12]
		NSP4-R660	5’-GTTGGGGTACCAGGGATTAA-3’		

* Genes abbreviations are based on the nomenclature suggested by authors cited in the reference column. NS = nonstructural; UTR = untranslated region; ORF = open reading frame; S4 = segment 4; NSP4 = nonstructural protein 4.

**Table 2:** Frequencies of single and multiple viral infections diagnosed in 270 samples.

Number of Viruses	FAdV-I	ANV	CAstV	IBV	ChPV	AReo	ARtV	Single and Multiple Positive Samples
1 virus	22	19	15	164	8	1	4	233/270 (86.3%)
2 viruses	3	6	7	14	1	0	3	17/270 (6.3%)
3 viruses	3	12	1	14	10	0	5	15/270 (5.6%)
4 viruses	0	4	3	3	2	0	4	4/270 (1.5%)
5 viruses	0	1	1	1	1	0	1	1/270 (0.4%)
Number of positive samples for each virus	28	42	27	196	22	1	17	-
% of samples positive for each virus (*n* = 333)	8.4%	12.6%	8.1%	58.9%	6.6%	0.3%	5.1%	-

**Table 3:** Enteric virus detection patterns from 270 samples in different types of organs from chicken commercial flocks.

Patterns	Virus Examined in 270 Digestive Organs								Number of Samples in Each Pattern (%)	
	FAdV-I	ChPV	CAstV	ANV	IBV	AReo		ARtV		
1	+	-	-	-	-	-	-		22	8.1%
2	+	-	-	+	-	-	-		1	0.4%
3	+	-	-	-	+	-	-		2	0.7%
4	+	+	-	-	+	-	-		2	0.7%
5	+	-	-	+	+	-	-		1	0.4%
6	-	-	-	+	-	-	-		19	7%
7	-	-	-	+	+	-	-		3	1.1%
8	-	-	+	+	-	-	-		1	0.4%
9	-	-	-	+	-	-	+		1	0.4%
10	-	+	-	+	+	-	-		7	2.6%
11	-	-	-	+	+	-	+		3	1.1%
12	-	+	-	+	-	-	+		1	0.4%
13	-	-	+	+	+	-	+		2	0.7%
14	-	+	+	+	-	-	+		1	0.4%
15	-	+	-	+	+	-	+		1	0.4%
16	-	+	+	+	+	-	+		1	0.4%
17	-	-	+	-	-	-	-		15	5.6%
18	-	-	+	-	+	-	-		6	2.2%
19	-	-	+	-	+	-	+		1	0.4%
20	-	-	-	-	+	-	-		164	60.7%
21	-	+	-	-	+	-	-		1	0.4%
22	-	-	-	-	+	-	+		2	0.7%
23	-	+	-	-	-	-	-		8	3%
24	-	-	-	-	-	-	+		4	1.5%
25	-	-	-	-	-	+	-		1	0.4%

**Table 4:** Results of number of positive samples according to the age of layer and breeder flocks.

Age of Flocks	Layers			Breeders		
	Number of Positive Samples		% of Samples Positive for Each Week of Age	Number of Positive Samples		% of Samples Positive for Each Week of Age
1–10 weeks	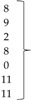	49	16.3%	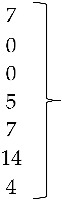	37	18.9%
11–20 weeks			18.4%			0%
21–30 weeks			4.1%			0%
31–40 weeks			16.3%			13.5%
41–50 weeks			0%			18.9%
>51 weeks			22.4%			37.8%
Not reported			22.4%			10.8%

**Table 5:** Number of positive samples according to the age of broilers.

Age of Flocks	Number of Positive Samples		% of Samples Positive for Each Day of Age
1–7 days	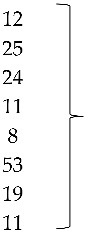	163	7.4%
8–14 days			15.3%
15–21 days			14.7%
22–28 days			6.7%
29–35 days			4.9%
36–42 days			32.5%
>43 days			11.7%
Not reported			6.7%

**Table 6:** Frequency of single and multiple virus infections and clinical signs according to the type of birds.

Item	Positive Samples with Single and Multiple Viral Infections					Clinical Signs				
	1 Virus	2 Viruses	3 Viruses	4 Viruses	5 Viruses	Respiratory Problems	Digestive Problems	Poor Production *	No Clinical Signs	Not Reported
Broilers (*n* = 163)	129 (55.4%)	14 (82.4%)	15 (100%)	4 (100%)	1 (100%)	12 (30.8%)	9 (100%)	55 (79.7%)	1 (20%)	86 (58%)
Layers (*n* = 49)	49 (21%)	0 (0%)	0 (0%)	0 (0%)	0 (0%)	23 (59%)	0 (0%)	2 (2.9%)	0 (0%)	24 (16.2%)
Breeders (*n* = 37)	34 (14.6%)	3 (17.6%)	0 (0%)	0 (0%)	0 (0%)	3 (7.7%)	0 (0%)	12 (17.4%)	4 (80%)	18 (12.2%)
Not reported (*n* = 21)	21 (9%)	0 (0%)	0 (0%)	0 (0%)	0 (0%)	1 (2.6%)	0 (0%)	0 (0%)	0 (0%)	20 (13.5%)
Number of positive samples (*n* = 270)	233	17	15	4	1	39	9	69	5	148
% of positive samples independently of chicken line	86.3%	6.3%	5.6%	1.5%	0.4%	14.4%	3.3%	25.6%	1.9%	54.8%

* Poor production was defined as culling, stunting, and mortality.

**Table 7:** Frequency of viruses found in different digestive organs.

Sample	FAdV-I	ChPV	CAstV	ANV	IBV	AReo	ARtV
Liver (*n* = 39)	19	3	1	1	14	0	2
Intestines (*n* = 89)	4	14	19	27	52	1	4
Pancreas (*n* = 25)	5	5	7	14	13	0	11
Caecal tonsils (*n* = 20)	0	0	0	0	20	0	0
Cloacal swabs (*n* = 97)	0	0	0	0	97	0	0
Total (viruses = 333)	28	22	27	42	196	1	17

**Table 8:** Frequency of viruses affecting birds according to the age of birds.

Chicken Lines	Age of Birds	FAdV-I		ChPV		CAstV		ANV		IBV		AReo		ARtV	
Layers	1–10 weeks	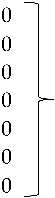	0	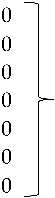	0	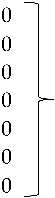	0	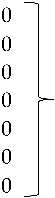	0	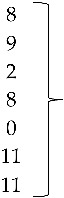	49	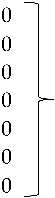	0	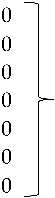	0
	11–20 weeks														
	21–30 weeks														
	31–40 weeks														
	41–50 weeks														
	>51 weeks														
	N/R														
Breeders	1–10 weeks	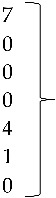	12	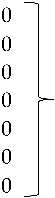	0	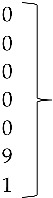	10	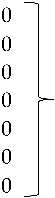	0	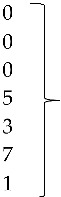	16	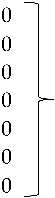	0	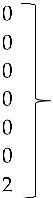	2
	11–20 weeks														
	21–30 weeks														
	31–40 weeks														
	41–50 weeks														
	>51 weeks														
	N/R														
Broilers	1–7 days	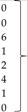	14	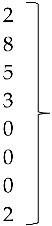	20	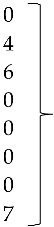	17	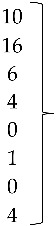	41	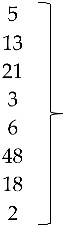	116	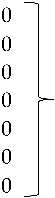	0	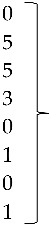	15
	8–14 days														
	15–21 days														
	22–28 days														
	29–35 days														
	36–42 days														
	>43 days														
	N/R														
Not reported neither age nor line		2		2		0		1		15		1		0	
Total viruses = 333 *		28		22		27		42		196		1		17	

* values based on 333 viruses detected. N/R= Not Reported age of birds.

**Table 9:** Frequencies of viruses detected in 12 Brazilian states.

Brazilian States—Samples Origin	Total Positive Samples *	% of Samples Positive for Each State	Virus Strains Examined in This Study **						
			FAdV-I	ChPV	CAstV	ANV	IBV	AReo	ARtV
Bahia	6	2.2%		3 (13.6) ^A^		6 (14.3)	5 (2.6)		
Ceara	5	1.9%		2 (9.1)		2 (4.8)	1 (0.5)		
Espirito Santo	3	1.1%					3 (1.5)		
Goias	13	4.8%					13 (6.6)		
Mato Grosso	11	4.1%					11 (5.6)		
Minas Gerais	50	18.5%		4 (18.2)	20 (74.1)	14 (33.3)	33 (16.8)		11 (64.7)
Paraiba	4	1.5%		1 (4.5)		3 (7.1)			
Pernambuco	9	3.3%	1 (3.6)	2 (9.1)		5 (11.9)	1 (0.5)		1 (5.9)
Piaui	1	0.4%					1 (0.5)		
Santa Catarina	7	2.6%	7 (25)						
São Paulo	82	30.4%	18 (64.3)	3 (13.6)		7 (16.7)	65 (33.2)		3 (17.6)
Not informed	79	29.3%	2 (7.1)	7 (31.8)	7 (25.9)	5 (11.9)	63 (32.1)	1 (100)	2 (11.8)
Total	270	100%	28 (100)	22 (100)	27 (100)	42 (100)	196 (100)	1 (100)	17 (100)

^A^ The numbers in parentheses represent the percentage values of each sample. The blanks in the table indicate that the sample showed negative results in the PCR assay. * Values calculated based on 270 samples received in the Avian Pathology Laboratory. ** Values calculated based on 333 viruses detected in the 270 samples.
